# The Long Intergenic Noncoding RNA *ARTA* Specifically Regulates MYB7 Nuclear Trafficking to Establish a Self-Reinforcing Circuit for ABA Response

**DOI:** 10.3390/plants15111596

**Published:** 2026-05-22

**Authors:** Zhengmin Tang, Jun Yang, Yanhang Chen, Yongdi Zhang, Jingjing Cai, Dong Wang, Reqing He, Youlin Zhu

**Affiliations:** 1 Institute of Advanced Agricultural Sciences, College of Life Science, Nanchang University, Nanchang 330031, China; 17861500710@163.com (Z.T.); yanhangchen@outlook.com (Y.C.); 17862186672@163.com (Y.Z.); caijingjing1234@163.com (J.C.); 2 Ministry of Education Key Laboratory of Crop Physiology, Ecology and Genetic Breeding, Jiangxi Agricultural University, Nanchang 330045, China; junyang2819@163.com

**Keywords:** lncRNA, feedback, nuclear trafficking, ABA

## Abstract

Long noncoding RNAs are involved in diverse biological processes in plants. Our recent study has revealed that an ABA-induced long intergenic noncoding RNA, *ARTA*, regulates both ABA and drought responses by blocking the nuclear import of a transcription factor, MYB7, through interacting with an importin β-like protein, SAD2. Here, we show that unlike MYB7, *ARTA* fails to disrupt interactions of SAD2 with the other two R2R3-MYB subgroup 4 members, MYB4 and MYB32. Consequently, the nuclear localizations of MYB4 and MYB32 remain unchanged upon alteration of *ARTA* expression. Furthermore, *ARTA* and MYB7 form a self-reinforcing feedback loop during *Arabidopsis* responses to ABA: ABA treatment induces *ARTA* expression, which in turn inhibits nuclear accumulation of MYB7, thereby deteriorating MYB7-mediated repression of *ARTA* and promoting *ARTA* production. This self-reinforcing feedback regulation elegantly integrates protein relocalization with transcriptional augmentation in the ABA response process, and provides a tunable molecular circuit for plant stress adaptation.

## 1. Introduction

Plants as sessile organisms have to cope with various environmental conditions. When plants encounter water deficit, the level of abscisic acid (ABA) increases and activates downstream signal transduction pathways [1]. Plants reduce water loss by regulating stomatal closure and initiate protective mechanisms to cope with drought stress [2]. Transcriptional regulation is a key step in ABA signal transduction, in which a large number of transcription factors are activated. These transcription factors then induce the expression of downstream functional genes (such as LEA protein-coding genes and antioxidant enzyme genes), ultimately enhancing the stress tolerance of plants [3]. In addition to protein-coding genes, increasing evidence shows that long noncoding RNAs (lncRNAs) are also widely involved in the regulation of plant ABA signaling pathways [4,5,6]. LncRNAs are RNAs with a length of more than 200 nucleotides and lack protein-coding potential [7]. According to its genomic location and transcription direction relative to protein-coding genes, lncRNA can be further divided into long intergenic noncoding RNAs (lincRNAs), natural antisense transcripts (NATs), long noncoding enhancer RNAs (lnc-eRNAs), precursor of small interfering RNA, etc. [8]. In plants, many lncRNAs have been annotated, but the molecular functions of most of them have not been fully elucidated [9]. LncRNAs, as important regulators of plant growth and stress responses, are able to function as molecular scaffolds for protein complex assembly, structural components in ribonucleoprotein complexes or chromatin architecture, and decoys or sponges for miRNAs, thereby coordinating various biological processes by modulating gene expression [8].

As scaffolds, lncRNAs can bring together multiple proteins to form functional ribonucleoprotein complexes. For instance, the lncRNA *DANA2* positively regulates drought tolerance by recruiting ERF84 to promote JMJ29-mediated histone demethylation [10]. Similarly, lncRNA *DANA1* interacts with the L1p/L10e family protein DIP1 to form an RNA–protein complex that recruits histone deacetylase HDA9 to regulate *CYP707A1* and *CYP707A2* expression [11]. As molecular sponges, lncRNAs can act as competing endogenous RNAs (ceRNAs), reducing the levels of free miRNAs and relieving the inhibitory effect of miRNAs on their target genes. A typical example in *Arabidopsis* is the lncRNA *IPS1*, which contains a sequence complementary to miR399 but has a mismatched loop structure at the cleavage site. This allows *IPS1* to sequester miR399 and prevent it from cleaving its target mRNA involved in phosphate homeostasis [12]. As structural components, lncRNAs can form R-loop structures with genomic DNA. In *Arabidopsis*, the lncRNA *APOLO* employs R-loop formation to recognize multiple genomic loci and, in combination with chromatin looping mechanisms, enables long-range transcriptional regulation of target genes [13].

Beyond their roles in chromatin and transcriptional regulation, plant lncRNAs also control protein localization and intracellular transport. For instance, in *Medicago truncatula*, the *enod40* RNA directly participates in the relocalization of its interacting protein, MtRBP1, from the nucleus to the cytoplasm [14]. In *Arabidopsis*, the nucleocytoplasmic transport protein SAD2 (an importin β-like protein) mediates the nuclear import of various proteins involved in diverse biological processes [4,15,16,17,18,19]. Our recent study has revealed that the ABA-induced lincRNA *ARTA* binds to SAD2 and affects the nuclear transport of MYB7, an R2R3-MYB subgroup 4 transcription factor that directly binds to the *ABI5* promoter and represses its transcription, thereby modulating *ABI5* expression [4,20]. Both *ARTA* and MYB7 have been shown to regulate ABA sensitivity and drought tolerance in *Arabidopsis* [4], underscoring the functional significance of the *ARTA*–MYB7 regulatory axis in ABA responses. Furthermore, accumulating evidence suggests that MYB transcription factors are widely involved in dynamic feedback loops that fine-tune signal responses in plants [21,22,23,24,25,26]. In addition to MYB7, SAD2 also facilitates the nuclear import of two other subgroup 4 members, MYB4 and MYB32 [17]. However, whether *ARTA* affects the interaction between SAD2 and MYB4 or MYB32 remains unclear.

In this study, we demonstrate that *ARTA* selectively disrupts the interaction between SAD2 and MYB7 both *in vitro* and *in vivo*, without affecting the binding of SAD2 to its homologs MYB4 and MYB32. Consistently, *ARTA* does not alter the nuclear localization of MYB4 or MYB32. Moreover, we discover that this regulation is bidirectional: MYB7 directly binds to the promoter of the *ARTA* and represses its transcription. Thus, our findings reveal a self-reinforcing feedback loop between *ARTA* and MYB7. ABA-induced *ARTA* blocks MYB7 nuclear import, which in turn relieves MYB7-mediated transcriptional repression of *ARTA*, thereby further promoting *ARTA* production and amplifying the ABA signal. Intriguingly, we also find that ABI5 represses *ARTA* transcription, providing a braking mechanism that prevents runaway amplification of this self-reinforcing loop and facilitates the restoration of homeostasis.

Taken together, these findings extend our previous understanding of *ARTA* function and provide a new molecular framework for ABA signaling regulation in plants.

## 2. Results

### 2.1. ARTA Did Not Impair Protein Interactions Among MYB4, MYB32, and SAD2

**Figure 1:**
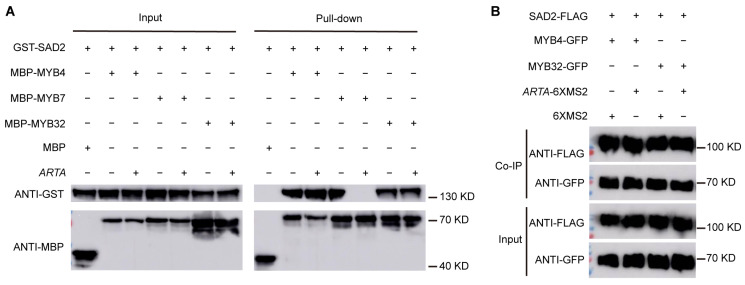
Examining effects of *ARTA* on interaction between SAD2 and two subgroup 4 R2R3-MYB members. (**A**) *In vitro* pull-down assay. *In vitro* transcribed *ARTA* (80 pmol) was incubated with GST-SAD2 and MBP-MYB4 or MBP-MYB32, and pulled down with amylose resin. Bound proteins were detected by immunoblotting with anti-GST and anti-MBP antibodies. (**B**) Co-IP assay of MYB4 and MYB32 with SAD2. MYB4-GFP or MYB32-GFP was co-expressed with SAD2-FLAG and MS2 or MS2-*ARTA* in *N. benthamiana* leaves. Protein extracts were immunoprecipitated with anti-GFP antibody, followed by immunoblotting with anti-FLAG antibody.

As reported, SAD2 influenced the nuclear transport of the R2R3-MYB subgroup 4 transcription factors MYB4, MYB7, and MYB32 [17]. Sequence alignment reveals that these three proteins share conserved R2R3 DNA-binding domains, the EAR motif, and a conserved SAD2-interacting SID motif at their C-termini, while notable sequence divergence exists outside these conserved regions [17]. Given their conserved SAD2-interacting motifs yet with regional sequence divergence, whether *ARTA* affects the SAD2-MYB4 or SAD2-MYB32 interaction remains unclear. To address this, we performed *in vitro* pull-down assays. These results showed that *ARTA* disrupted the SAD2-MYB7 interaction, consistent with the previous finding, but did not block the formation of either the SAD2-MYB4 or the SAD2-MYB32 complex under identical experimental conditions (Figure 1A).

To validate these *in vitro* findings in a cellular context, we performed co-immunoprecipitation (Co-IP) assays in *Nicotiana benthamiana* leaves transiently co-expressing the proteins. The Co-IP results clearly demonstrated that the existence of *ARTA* RNA did not influence the interaction of SAD2 with MYB4 or MYB32, respectively (Figure 1B). These findings further substantiated that *ARTA* does not impair the binding affinity between SAD2 and MYB4 or MYB32 *in vivo*.

Taken together, the above results prove that *ARTA* selectively disrupts the SAD2-MYB7 interaction, but does not affect the closely related SAD2-MYB4 and SAD2-MYB32 complexes. Therefore, we conclude that *ARTA* functions as a precise molecular disruptor specifically targeting the SAD2-MYB7 axis.

### 2.2. ARTA Did Not Alter the Nuclear Localizations of Both MYB4 and MYB32

**Figure 2:**
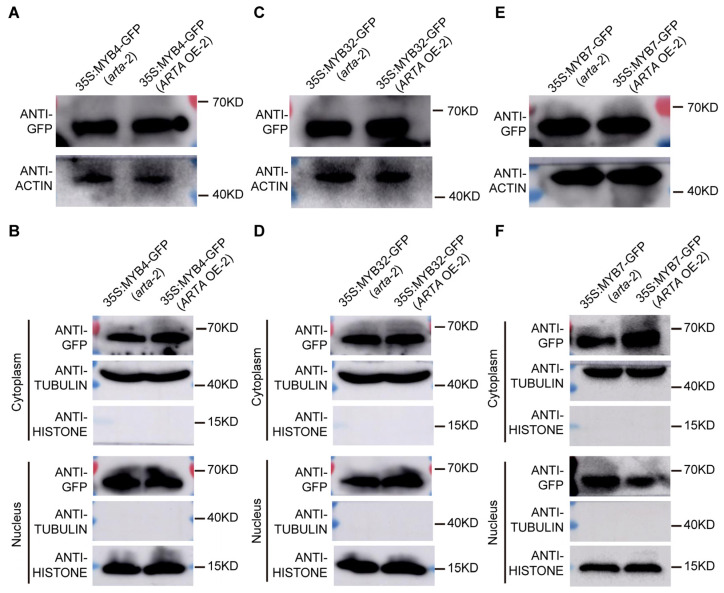
Examining effects of *ARTA* on nuclear localization of MYB4, MYB7, and MYB32. (**A**) *35S*:MYB4-GFP was transfected into *arta-2* and *ARTA* OE-2 protoplasts and the total protein levels of MYB4-GFP were detected. ACTIN was used as a loading control. (**B**) Immunoblot analyses showing the nucleus and cytoplasmic distributions of MYB4 protein in *arta-2* and *ARTA* OE-2. HISTONE and TUBULIN were used as a nuclear and cytoplasmic marker, respectively. (**C**) *35S*:MYB32-GFP was transfected into *arta-2* and *ARTA* OE-2 protoplasts and the total protein levels of MYB32-GFP were detected. ACTIN was used as a loading control. (**D**) Immunoblot analyses showing the nucleus and cytoplasmic distributions of MYB32 protein in *arta-2* and *ARTA* OE-2. HISTONE and TUBULIN were used as a nuclear and cytoplasmic marker, respectively. (**E**) *35S*:MYB7-GFP was transfected into *arta-2* and *ARTA* OE-2 protoplasts and the total protein levels of MYB7-GFP were detected. ACTIN was used as a loading control. (**F**) Immunoblot analyses showing the nucleus and cytoplasmic distributions of MYB7 protein in *arta-2* and *ARTA* OE-2. HISTONE and TUBULIN were used as a nuclear and cytoplasmic marker, respectively.

To investigate whether *ARTA* affected the nuclear translocation of MYB4 and MYB32, we performed transient expression in mesophyll protoplasts. Protoplasts isolated from both *arta-2* and *ARTA*-overexpressing (*ARTA* OE) plants were transfected separately with constructs expressing MYB4-GFP, MYB7-GFP, or MYB32-GFP driven by the *35S* promoter. First, we confirmed by Western blot analysis that no apparent differences in the protein levels of MYB4-GFP, MYB7-GFP, and MYB32-GFP were observed between *arta-2* and *ARTA* OE plants (Figure 2A,C,E).

Subsequently, to determine whether *ARTA* influences the nucleocytoplasmic partitioning of MYB4 and MYB32, subcellular fractionation and immunoblotting experiments were performed to quantitatively assess their nuclear versus cytoplasmic distribution. Using histone and tubulin as rigorous indicators for the nuclear and cytoplasmic fractions, respectively, we observed that MYB7-GFP nuclear accumulation was markedly reduced in the *ARTA* OE plant compared with *arta-2*, consistent with our previous finding [4]. In contrast, no significant difference in the nuclear accumulation of either MYB4-GFP or MYB32-GFP was detected between the *arta* mutant and *ARTA* OE plants (Figure 2B,D,F). The results indicated that *ARTA* effectively regulates the nuclear import of MYB7 but exerts no detectable effect on the nuclear translocation of its paralogs, MYB4 and MYB32.

### 2.3. MYB7 Repressed ARTA Expression by Directly Binding to Its Promoter

**Figure 3:**
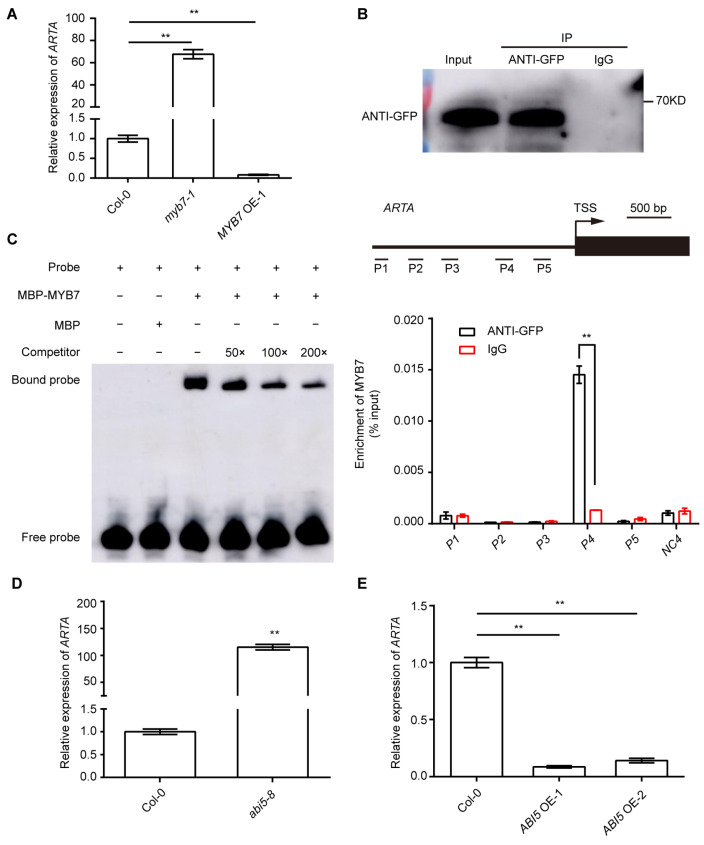
MYB7 regulated *ARTA* expression. (**A**) Analysis of *ARTA* transcript levels in 4-day-old Col-0, *myb7-1* mutant, and MYB7-overexpressing plants. Data are shown as mean ± SD (*n* = 3). Asterisks indicate statistically significant differences as determined by Student’s *t*-test (** *p* < 0.01). (**B**) (**Top**) Western blot analysis of MYB7-GFP protein levels in input and immunoprecipitated (IP) fractions. (**Middle**) Schematic diagram of the *ARTA* promoter region. Positions of five primer pairs (P1–P5) used for ChIP-qPCR are indicated relative to the transcription start site (TSS). (**Bottom**) ChIP-qPCR analysis of MYB7 binding at the promoters of *ARTA* in *MYB7* OE plants. *NC4* served as the negative control. Data are shown as mean ± SD (*n* = 3). Asterisks indicate statistically significant differences as determined by Student’s *t*-test (** *p* < 0.01). (**C**) EMSA of MYB7 binding the *ARTA* promoter. Biotin-labeled 36-nucleotide DNA probe derived from the P4 region of the *ARTA* promoter was incubated with recombinant MBP-MYB7 protein. Unlabeled probe was used as a competitor. (**D**) Quantitative measurement of the transcript levels of *ARTA* in 4-day-old Col-0 and *abi5-8* plants. Data are shown as mean ± SD (*n* = 3). Asterisks indicate statistically significant differences as determined by Student’s *t*-test (** *p* < 0.01). (**E**) Quantitative measurement of the transcript levels of *ARTA* in 4-day-old Col-0 and *ABI5* OE plants. Data are shown as mean ± SD (*n* = 3). Asterisks indicate statistically significant differences as determined by Student’s *t*-test (** *p* < 0.01).

Given that *ARTA* specifically inhibited MYB7 nuclear import, we next asked whether MYB7 reciprocally regulates *ARTA* expression, thereby forming a feedback loop. The RT-qPCR results showed that *ARTA* transcript levels were significantly upregulated in the *myb7* mutant and downregulated in *MYB7* OE plants compared to the wild type, indicating that MYB7 acted as a transcriptional repressor of *ARTA* (Figure 3A). To determine whether MYB7 directly binds to the *ARTA* promoter, we performed a chromatin immunoprecipitation (ChIP)-qPCR assay using *35S*:MYB7-GFP transgenic *Arabidopsis* seedlings. The results showed that MYB7 was significantly enriched at the P4 region of the *ARTA* promoter (Figure 3B). Next, an electrophoretic mobility shift assay (EMSA) was carried out, and the result further confirmed that MYB7 directly binds to the P4 region of the *ARTA* promoter *in vitro* (Figure 3C). These results indicate that MYB7 directly binds to the *ARTA* promoter to repress its transcription, thereby forming a dual-negative feedback loop with *ARTA*.

Given that MYB7 directly represses *ABI5* transcription and *ARTA* relieves this repression by blocking MYB7 nuclear import [4], we next examined whether ABI5 in turn regulates *ARTA*. RT-qPCR analysis showed that *ARTA* transcript levels were significantly upregulated in the *abi5-8* mutant and downregulated in ABI5-overexpressing (*ABI5* OE) plants compared to Col-0 (Figure 3D,E), indicating that ABI5 also functions as a transcriptional repressor of *ARTA*.

## 3. Discussion

The precise spatial and temporal control of transcription factor activity is fundamental to eukaryotic gene regulation. In plants, this is often achieved by modulating the nucleocytoplasmic partitioning of key transcriptional regulators [15,27,28,29,30,31]. Previously, we found that lincRNA *ARTA* could regulate *ABI5* expression during plant responses to ABA by regulating MYB7 nuclear trafficking [4]. MYB7 belongs to the R2R3-MYB subgroup 4, and SAD2 has been validated to be involved in the nuclear import of subgroup 4 R2R3-MYB TFs except for MYB3 [17]. However, the effects of *ARTA* on the nuclear translocation of MYB4 and MYB32 remain unclear. Here, our results showed that *ARTA* did not impair protein interactions among MYB4, MYB32, and SAD2 (Figure 1), and *ARTA* did not alter the nuclear localizations of either MYB4 or MYB32 as well (Figure 2).

The differential effects of *ARTA* on different SAD2 substrates prompted us to uncover the underlying molecular basis. Using structural bioinformatics analysis with PISA, we revealed significant differences in the thermodynamic properties of these complexes. The solvation free energy increment (ΔiG) upon formation of the SAD2-MYB7 complex interface was −9.6 kcal/mol, which is substantially higher than that of SAD2-MYB4 (−23.8 kcal/mol) and SAD2-MYB32 (−28.7 kcal/mol) (Figure S1). This indicated that the interaction between SAD2 and MYB7 is relatively weak and the interface is inherently unstable. Given this marked thermodynamic disparity, we propose that such weak binding renders the SAD2-MYB7 complex uniquely susceptible to disruption by *ARTA.* In contrast, the deeply favorable binding energies of MYB4 and MYB32 likely confer resistance to such interference. Even if *ARTA* binds to a fraction of SAD2, the high-affinity binding of MYB4 and MYB32 enables them to efficiently compete for the residual SAD2 pool, sustaining unperturbed nuclear import. Future structural studies will be essential to further elucidate the precise molecular mechanism by which *ARTA* distinguishes between different SAD2–substrate complexes.

**Figure 4:**
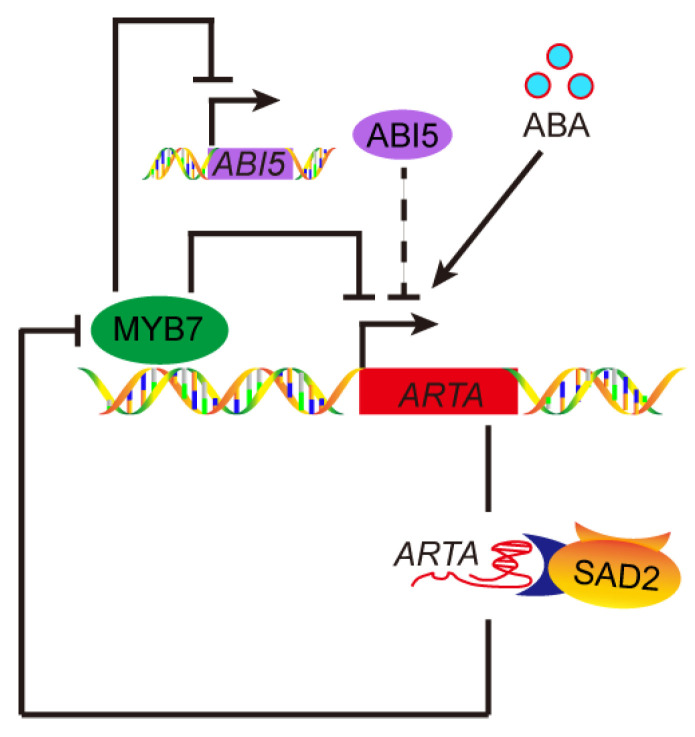
A model for *ARTA*-regulated ABA signaling. ABA-induced lincRNA *ARTA* binds to SAD2, disrupting the interaction between SAD2 and MYB7, thereby reducing nuclear-localized MYB7 protein levels [4]. This relieves MYB7-mediated repression of the *ARTA* promoter, thereby driving further *ARTA* synthesis. This establishes a self-reinforcing feedback loop between *ARTA* and MYB7. On the other hand, the reduction in nuclear MYB7 attenuates its repression of *ABI5*, promoting *ABI5* transcription [4]. Subsequently, ABI5 suppresses *ARTA* expression through negative feedback, ultimately dampening the *ARTA* response and restores homeostasis. Blunt-ended arrows indicate an inhibitory effect, the two right-angled arrows indicate the direction of transcription of the *ABI5* and *ARTA* genes, the dashed line indicates whether ABI5 directly inhibits *ARTA* remains unknown and the solid arrow indicates ABA induction.

Feedback regulation is a key mechanism for balancing diverse physiological processes in organisms and enables rapid and reversible adaptive responses in plant growth, development, and environmental adaptation [24,25,26,32,33,34,35,36,37,38]. Our study revealed that the MYB7 protein binds to the promoter region of *ARTA* and represses its expression (Figure 3A). This establishes a critical self-reinforcing feedback loop: an initial ABA signal induces *ARTA* transcription [4]. Once *ARTA* accumulates and blocks MYB7 nuclear import [4], nuclear MYB7 levels decrease, which relieves the repression of the *ARTA* locus and further promotes *ARTA* transcription. Furthermore, we found that ABI5 represses *ARTA* transcription (Figure 3D,E). Based on these discoveries we propose a dynamic regulatory model (Figure 4). Under initial stress conditions, the *ARTA*-MYB7 double negative feedback loop is rapidly activated to amplify the ABA signal. As nuclear MYB7 is depleted by *ARTA*, its repression of *ABI5* is relieved, allowing ABI5 to accumulate [4]. Subsequently, ABI5 accumulates and provides a braking effect by inhibiting *ARTA* transcription, thereby preventing overactivation and promoting the restoration of homeostasis. Through this *ARTA*-MYB7-ABI5 regulatory module, plants achieve efficient and reversible stress adaptation. This regulatory mechanism warrants further refinement with respect to its spatial, temporal, and signal intensity-dependent dynamics in future studies.

In the current study, we revealed a double negative feedback loop, in which the lincRNA *ARTA* specifically inhibits the nuclear transport of the MYB7 protein. This inhibition relieves the MYB7-mediated transcriptional repression of *ARTA*, thereby establishing a self-reinforcing cycle that enables a rapid and amplified response to ABA signals. However, this circuit is further modulated by an ABI5-mediated braking mechanism, ensuring that the ABA response remains controlled. Our findings extend the understanding of *ARTA*’s regulatory mechanism and further enrich the feedback regulation networks for ABA signaling in plants.

## 4. Materials and Methods

### 4.1. Plant Materials and Growth Conditions

All transgenic lines used in this study were in the Col-0 background. The mutants *myb7-1* (SALK_020256) and *abi5-8* (SALK_013163), obtained from the Arabidopsis Biological Resource Center (ABRC, Ohio State University, Columbus, OH, USA), have been described previously [4,39,40]. The *MYB7* OE line was generated by expressing the complementary DNA encoding full-length *MYB7* driven by the 35S promoter, fused with a GFP tag, as previously described [4]. The *ABI5* OE line was generated by expressing the complementary DNA encoding full-length *ABI5* driven by the 35S promoter, fused with an HA tag, as previously described [4]. *Arabidopsis* seeds were sterilized with 70% ethanol for 10 min, followed by six to seven rinses with sterile distilled water. Subsequently, the seeds were sown in 1/2 MS medium, layered for 3 days in the darkness of 4 °C, and grown under the illumination of 22 °C for 16 h light/8 h dark.

### 4.2. RT-qPCR and Western Blotting

Total RNA was extracted using the TRIzol reagent (Ambion, Austin, TX, USA, Cat# 15596018), and first-strand cDNA was synthesized with the TransScript One-Step gDNA Removal and cDNA Synthesis SuperMix (TransGen Biotech, Beijing, China, Cat# AT311-03). RT-qPCR was performed on a Bio-Rad CFX96 system using the SYBR qPCR Master Mix (Vazyme, Nanjing, China, Cat# Q711-02). *UBQ3* was used as the reference gene for normalization. Three independent biological samples were considered. Student’s *t* test was performed considering two-tailed samples of different variance. Primer sequences are listed in Table S1.

Protein extracts were resolved on 12% SDS-PAGE gels (Thermo Fisher Scientific, Waltham, MA, USA, Cat# XP00120BOX) and transferred to nitrocellulose membranes (GE Healthcare, Chicago, IL, USA, Cat# 9004700100). For immunoblotting, the following primary antibodies were used: anti-MBP (Abmart, Shanghai, China, Cat# M20051), anti-GST (Abmart, Shanghai, China, Cat# M20007), anti-GFP (BBI Life Sciences, Shanghai, China, Cat# D110008-0200), anti-FLAG (Abmart, Shanghai, China, Cat# M20008), anti-ACTIN (Abmart, Shanghai, China, Cat# M20009), anti-Tubulin (Abmart, Shanghai, China, Cat# M20045F), and anti-Histone (Abmart, Shanghai, China, Cat# P30266F).

### 4.3. Recombinant Proteins and In Vitro Protein Pull-Down Assay

The full-length cDNAs of MYB4, MYB32, and MYB7 were introduced into the vector pMSCG7-MBP, respectively. The full-length cDNA of SAD2 was introduced into the vector pGEX-6P-3 (GST). After that the resultant MBP-MYB4, MBP-MYB32, MBP-MYB7, and GST-SAD2 were separately transformed into the *Escherichia coli* (BL21 (DE3)) cells. The pull-down assay was performed as previously described [4]. In brief, 500 μg of GST-SAD2 was mixed with 500 μg of MBP, MBP-MYB4, MBP-MYB32, or MBP-MYB7 and the volume was adjusted to 1 mL with TGH buffer (50 mM HEPES pH 7.5, 150 mM NaCl, 1.5 mM MgCl_2_, 1 mM EGTA pH 7.5, 1% Triton X-100, 5% glycerol, 1 mM PMSF, and complete protease inhibitor cocktail (Roche, Basel, Switzerland, Cat# 04693132001)). The mixture was incubated for 1 h at 4 °C. Subsequently, 65 µL of amylose resin (NEB, Ipswich, MA, USA, Cat# E8021S) was added and incubated for an additional 1 h, followed by four washes with TGH buffer. The bound proteins were analyzed on 10% SDS-PAGE gels, and then subjected to immunoblotting.

### 4.4. Chromatin Immunoprecipitation

ChIP was performed as described previously with minor modifications [4]. Approximately 3 g of *35S*: MYB7-GFP seedlings were cross-linked *in vivo*, and cell nuclei were purified and extracted through sonication. The resulting supernatant was immunoprecipitated with mouse IgG1 (Cell Signaling Technology, Danvers, MA, USA, Cat# 5415S) or anti-GFP antibody (Roche, Basel, Switzerland, Cat# 11814460001) at 4 °C for 4h on a rotation mixer. After reverse cross-linking and proteinase K (Merck Millipore, Billerica, MA, USA, Cat# 539480) digestion, DNA was extracted with phenol–chloroform and precipitated with ethanol. The primer sequences used for ChIP-qPCR are listed in Table S1.

### 4.5. Electrophoretic Mobility Shift Assay

The EMSA was performed using the LightShift EMSA Optimization and Control Kit (Thermo Fisher Scientific, Waltham, MA, USA, Cat# 20148X) as described previously [4]. The biotin 5′-end-labeled DNA fragment (listed in Table S1) was synthesized, annealed, and used as the DNA probe, while the corresponding unlabeled DNA fragment served as the specific competitor. For the binding reaction, recombinant MBP-MYB7 proteins were incubated with 20 fmol of biotin-labeled probe in 20 μL of binding buffer (10 mM Tris-HCl pH 7.5, 50 mM KCl, 1 mM DTT, 2.5% glycerol, 5 mM MgCl_2_, 50 ng/μL poly (dI-dC)) at room temperature for 20 min. For competition assays, a 50-, 100-, or 200-fold molar excess of unlabeled probe was added to the reaction mixture prior to the addition of the labeled probe. The reaction products were separated on a native 6% polyacrylamide gel in 0.5× TBE buffer at 100 V for 60 min at 4 °C. Subsequently, the separated DNA–protein complexes were then transferred to a positively charged nylon membrane and cross-linked using a UV cross-linker. Biotin-labeled DNA was detected by chemiluminescence.

### 4.6. Co-IP Assay

Co-IP assays were performed as described previously with small modifications [41]. Briefly, Agrobacterium strains harboring *35S*: MYB4-GFP, *35S*: MYB32-GFP, *35S*: SAD2-FLAG, and *35S*: MS2 or *35S*: MS2-*ARTA* constructs described above were transiently expressed in leaves of *N. benthamiana*. After 48 h of infiltration, leaf tissues were harvested and protein extracts were incubated with anti-GFP antibody (Roche, Basel, Switzerland, Cat# 11814460001) to pull down the target proteins. After washing the magnetic beads, the immunoprecipitated proteins were resolved by SDS-PAGE and subsequently probed using anti-GFP (BBI Life Sciences, Shanghai, China, Cat# D110008-0200) or anti-FLAG (Abmart, Shanghai, China, Cat# M20008) antibodies.

### 4.7. Nuclear–Cytoplasmic Fractionation

Nuclear–cytoplasmic fractionation was carried out using protoplasts isolated from 3-week-old Arabidopsis plants of the *arta-2* and *ARTA* OE-2 lines, following a previous description [42]. The full-length MYB4 and MYB32 coding sequences were cloned into the vectors PA7-YFP, respectively. All constructs were transformed into Arabidopsis protoplasts by PEG-mediated transfection [4]. Subsequently, extracts were separated on 12% SDS-PAGE gels. After electrophoretic transfer to membranes, immunoblotting was performed using anti-GFP (BBI Life Sciences, Shanghai, China, Cat# D110008-0200), anti-Tubulin (Abmart, Shanghai, China, Cat# M20045F), and anti-Histone (Abmart, Shanghai, China, Cat# P30266F) antibodies.

## Data Availability

The original contributions presented in this study are included in the article/Supplementary Material. Further inquiries can be directed to the corresponding author.

## Supplementary Materials

The following supporting information can be downloaded at: https://www.mdpi.com/article/10.3390/plants15111596/s1. Figure S1: PISA-calculated interaction parameters for SAD2-MYB complexes. Table S1: Primers and probes used in this study.
