# Supplementary material for: The Long Intergenic Noncoding RNA ARTA Specifically Regulates MYB7 Nuclear Trafficking to Establish a Self-Reinforcing Circuit for ABA Response
Source: Plants (Basel). 2026 May 22;15(11):1596. doi: 10.3390/plants15111596 (PMC13259478; doi:10.3390/plants15111596)
Supplement: Supplementary file 1 [file plants-15-01596-s001.zip › Figure S1.pdf]

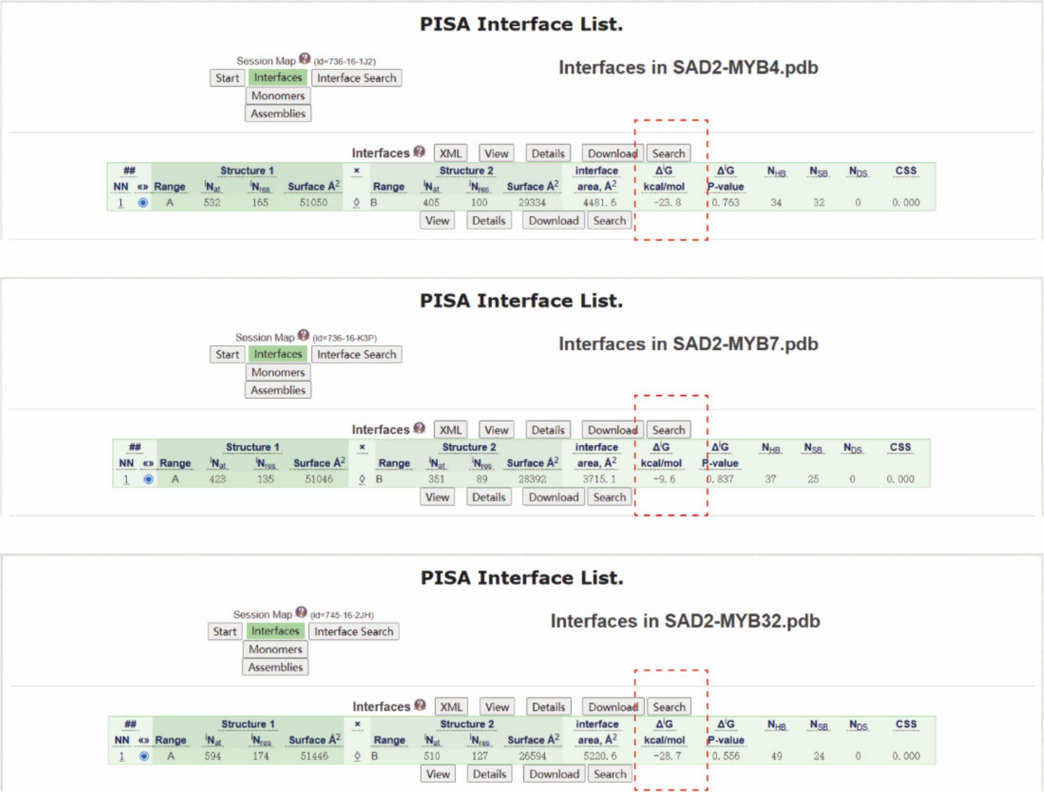

Figure S1. **PISA-calculated interaction parameters for SAD2-MYB complexes.** Thermodynamic and structural parameters of the SAD2-MYB complexes were analyzed using the PISA server. The solvation free energy gain (ΔiG) upon interface formation indicates the stability of the interaction, with more negative values representing tighter binding. The substantially higher ΔiG value (less negative) for the SAD2-MYB7 complex (-9.6 kcal/mol) compared to SAD2-MYB4 (-23.8 kcal/mol) and SAD2-MYB32 (-28.7 kcal/mol) suggests that the SAD2-MYB7 interface is intrinsically less stable and therefore more susceptible to disruption by *ARTA*. Interface area (Å²), number of hydrogen bonds (N_{HB}), and number of salt bridges (N_{SB}) are also shown.
